# Supplementary material for: Contrasting Responses of Multispatial Soil Fungal Communities of Thuja sutchuenensis Franch., an Extremely Endangered Conifer in Southwestern China
Source: Microbiol Spectr. 2022 Jun 23;10(4):e00260-22. doi: 10.1128/spectrum.00260-22 (PMC9431436; doi:10.1128/spectrum.00260-22)
Supplement: Supplemental file 1 — Supplemental material. Download spectrum.00260-22-s0001.pdf, PDF file, 1.3 MB [file spectrum.00260-22-s0001.pdf]

Contrasting responses of multi-spatial soil fungal communities of *Thuja sutchuenensis* Franch., an extremely endangered conifer in southwestern China

You-wei Zuo[1,2], Ping He[3], Jia-hui Zhang[1,2], Weng-qiao Li[1,2], Deng-hao Ning[1,2], Yu-lian Zeng[1,2], Ying Yang[1,2], Chang-ying Xia[1,2], Huan Zhang[1,2], Hong-ping Deng[1,2,3*]

[1] Center for Biodiversity Conservation and Utilization, School of Life Sciences, Southwest University, 400715, Beibei, Chongqing, China

[2] Chongqing Key Laboratory of Plant Resource Conservation and Germplasm Innovation, Institute of Resources Botany, School of Life Sciences, Southwest University, 400715, Beibei, Chongqing, China

[3] Chongqing Academy of Science and Technology, Low Carbon and Ecological Environment Protection Research Center, 401123, Liangjiang New Area, Chongqing, China

[*] Corresponding author

Hong-ping Deng; E-mail: denghp@swu.edu.cn; Phone number: +86 13883395687

Methods

Soil physical and chemical properties

Soil pH. A total of 10 g soil sample was passed through a 1 mm sieve and then added with 25 mL distilled water. The above solution was allowed to stand for 30 min, and the suspension was measured for pH with a PH meter (PHS-2F).

Soil organic matter. 0.1-0.5 g (recorded the accurate value) soil sample passed through a 60-mesh sieve (< 0.25 mm), and then added into 10 mL 0.36 mol/L potassium dichromate-sulfuric acid solution, shaken well, boiled at 185-190°C. After cooling, the above solution was added with 3-4 drops of phenanthroline, followed by a standard solution of ferrous sulfate ($FeSO_4$) (0.2 mol/L). Recorded the content of the added $FeSO_4$, and finally calculated the soil organic matter regarding the previous method (Bao, 2000).

Soil water content. The fresh soil sample was accurately weighed and baked for 12h in an oven preheated to 105°C. Subsequently, the treated soil sample was moved into a dryer to cool to room temperature (30 min) and weighed immediately. The calculated formula was the same as the previous method (Bao, 2000).

Soil nutrients. Soil total nitrogen was determined by semimicro-Kjeldahl (KDY-9820) digestion. Soil total phosphorus was determined colorimetrically using the molybdate method. Soil total potassium was measured by flame spectrophotometry. Soil available nitrogen was measured using the potassium dichromate external heating method. Soil available phosphorus was determined using the molybdenum blue method. Soil available potassium was determined by flame photometry.

43

Table S1. The dominant tree species at five elevational gradients

Elevation (m)	Dominant tree species (*n*)	Species diversity index			
		Shannon	Simpson	Gleason	Pielou
980	*Thuja sutchuenensis* Franch. (28), *Quercus semicarpifolia* Smith (28), *Cyclobalanopsis multinervis* Cheng et T. Hong (17)	3.818	0.93	3.171	1.297
1073	*Thuja sutchuenensis* Franch. (26), *Quercus semicarpifolia* Smith (19), *Cyclobalanopsis myrsinaefolia*(Bl.)Oerst. (15)	2.06	0.735	1.52	1.058
1512	*Thuja sutchuenensis* Franch. (15), *Quercus semicarpifolia* Smith (6), *Quercus phillyraeoides* A. Gray (3)	3.599	0.908	2.67	1.298
1658	*Pinus armandi* Franch. (20), J*uglans cathayensis* Dode (17), *Carpinus cordata* Bl.var. chinensis Franch. (9)	2.869	0.858	2.67	1.035
2119	*Quercus semicarpifolia* Smith (9), *Elaeagnus henryi* Warb. (7), *Pinus massoniana* Lamb. (6)	2.292	0.759	1.52	1.178

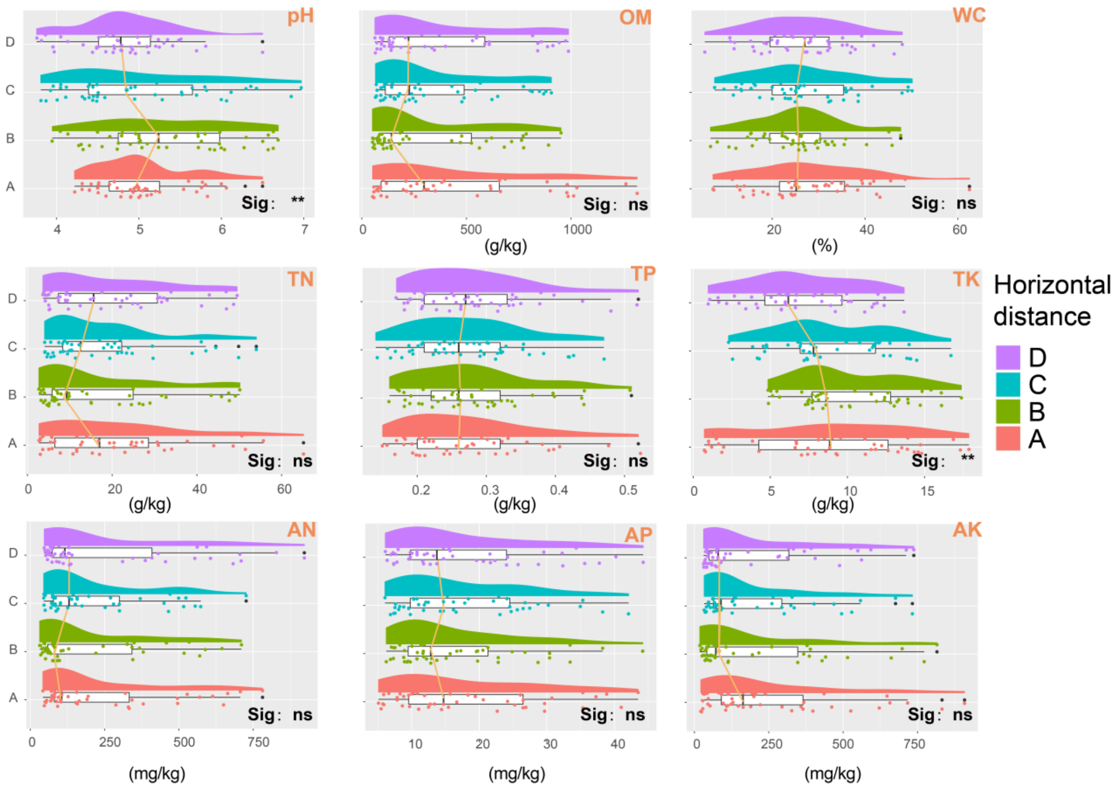

Fig. S1. Raincloud plot showing soil properties at different horizontal distances. The half violin diagram (cloud) shows the kernel density of the data distribution, and the scatter diagram (rain) shows the degree of dispersion. The rain-cloud plot also includes a box plot (umbrella) and lines (thunder) that link the median of different groups. Abbreviations: organic matter (OM); water content (WC); total nitrogen (TN); total phosphorus (TP); total potassium (TK); available nitrogen (AN); available phosphorus (AP); available potassium (AK). Four sites were selected as horizontal soil collection points according to the distance between sampling points and the base of the trunk: near the base (A), the center of the crown (B), the edge of the crown (C), and the bare area outside the crown (D). ns or ** indicated non-significant differences or p <0.01.

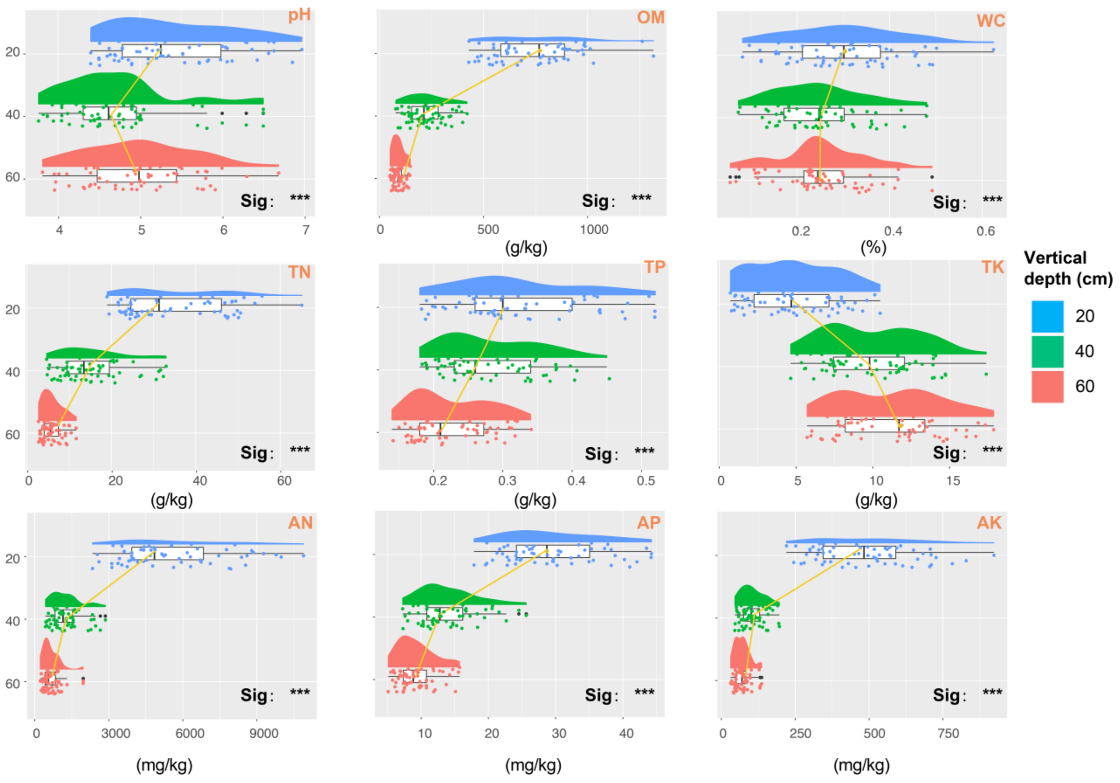

Fig. S2. Raincloud plot showing soil properties at different vertical depths. The half violin diagram (cloud) shows the kernel density of the data distribution, and the scatter diagram (rain) shows the degree of dispersion. The rain-cloud plot also includes a box plot (umbrella) and lines (thunder) that link the median of different groups. Abbreviations: organic matter (OM); water content (WC); total nitrogen (TN); total phosphorus (TP); total potassium (TK); available nitrogen (AN); available phosphorus (AP); available potassium (AK). Three sites were selected as vertical samples with depths of 20 cm, 40 cm, and 60 cm. *** indicated significant differences $p < 0.001$.

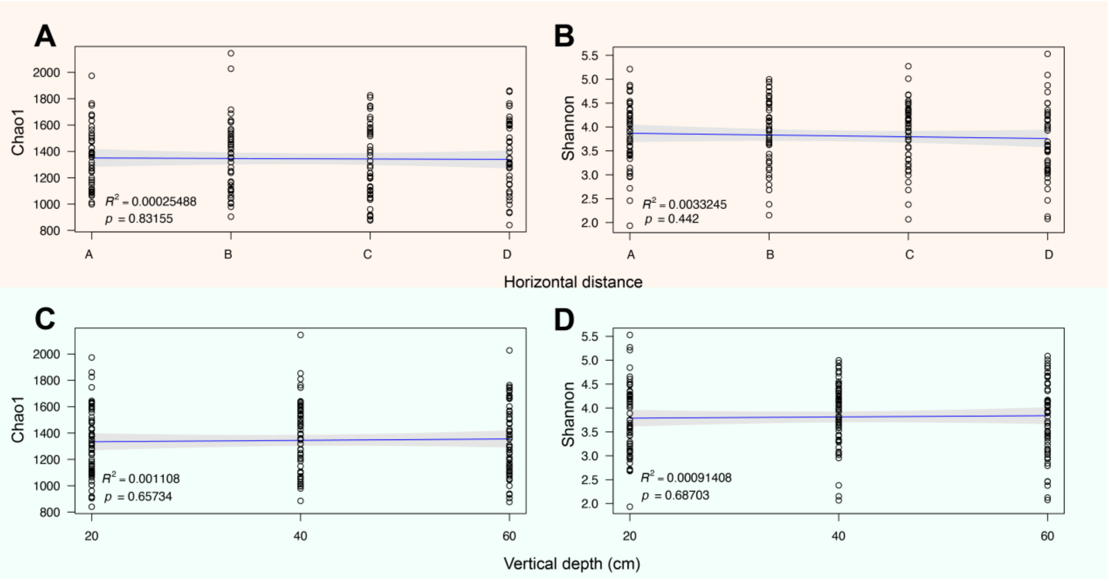

Fig. S3. Least-squares linear regression of fungal alpha-diversity indexes (Chao1 and Shannon) according with horizontal distances (A & B) and vertical depths (cm) (C & D).

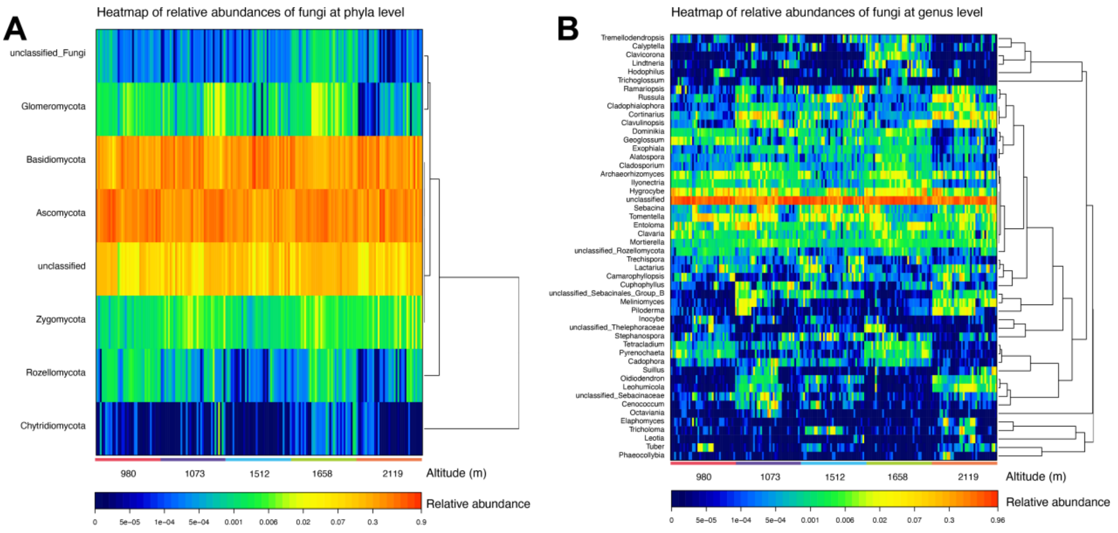

Fig. S4. Heatmap of relative abundances of fungi at phyla (A) and genus (B) levels. The color depth of the heat map indicates the abundance of fungi. Red indicates high expression and blue indicates low expression.

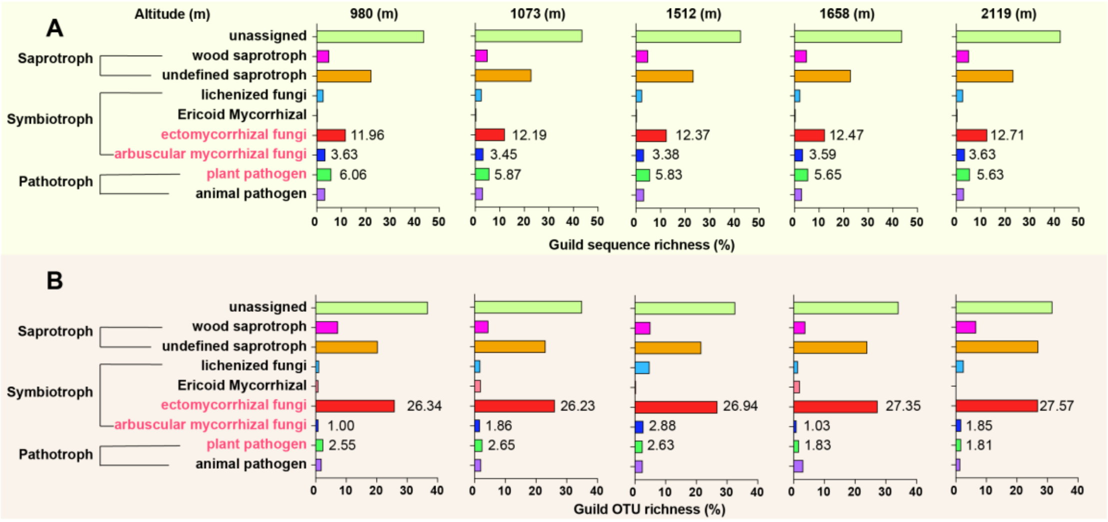

Fig. S5. Guild assignments for the yielded OTUs using FUNGuild. (**A & B**) Proportion of sequence richness and proportion of OTU richness assigned to guilds. OTUs and sequences not assigned to guilds were placed into the unassigned group.

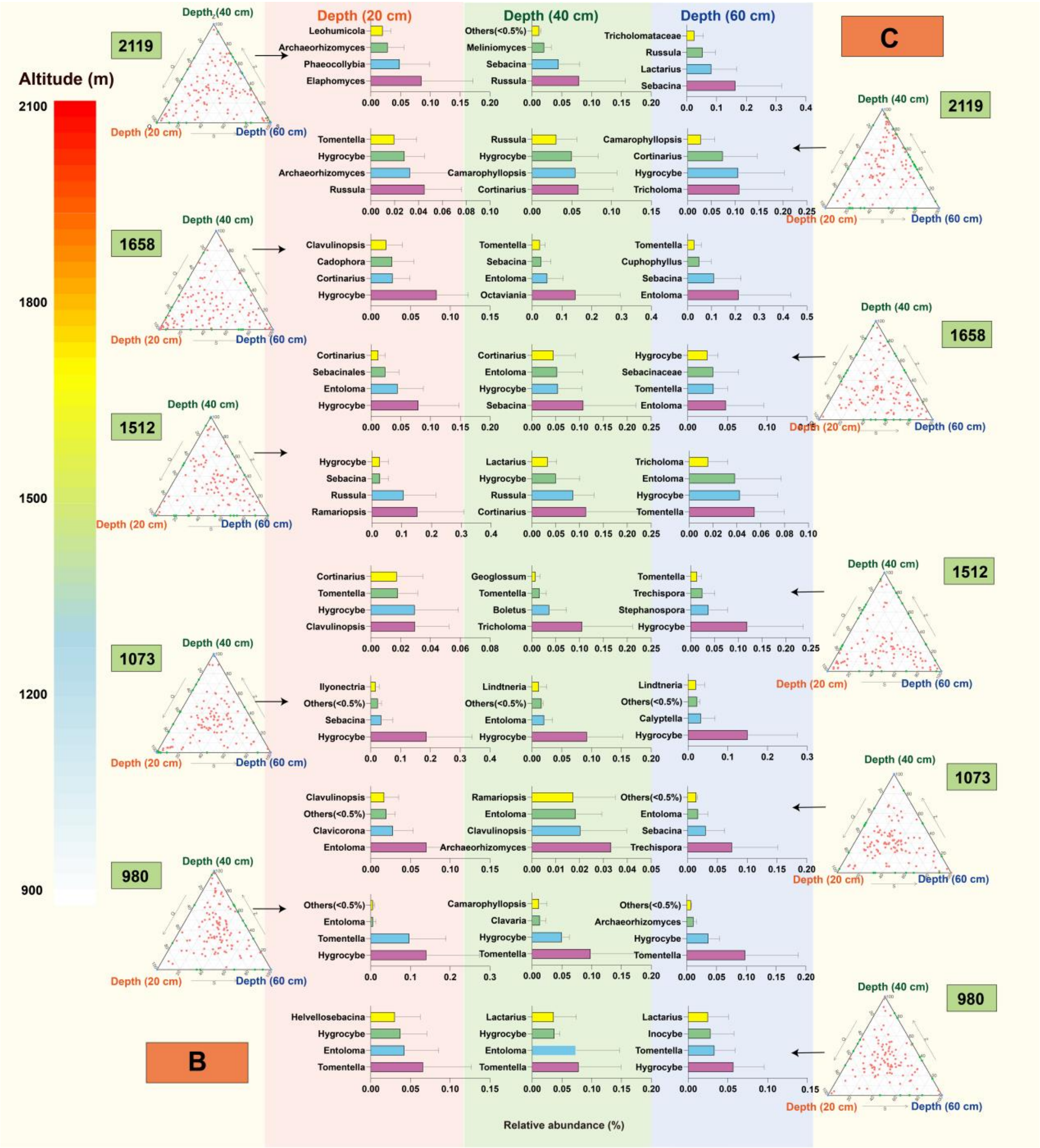

Fig. S6. Taxonomic distribution of fungal taxa (genera) accounting for community differentiation among different spatial variations. (B~left panel & C~right panel) represent the soil samples between base of the trunk (A) and bare area (D). Ternary plots display the distributions of the identified differentiation taxa. Each point represents one OTU. The most abundant four genera were presented in barplots. The colors of points indicate the OTUs dramatically enriched among distinct depths.

92
